# GPS driving: a digital biomarker for preclinical Alzheimer disease

**DOI:** 10.1186/s13195-021-00852-1

**Published:** 2021-06-14

**Authors:** Sayeh Bayat, Ganesh M. Babulal, Suzanne E. Schindler, Anne M. Fagan, John C. Morris, Alex Mihailidis, Catherine M. Roe

**Affiliations:** 1 Institute of Biomedical Engineering, University of Toronto, 550 University Avenue, Toronto, ON M5G 1X5 Canada; 2 KITE Research Institute, Toronto Rehabilitation Institute, Toronto, ON Canada; 3 Charles F. and Joanne Knight Alzheimer Disease Research Center, Washington University School of Medicine, St. Louis, MO USA; 4 Department of Neurology, Washington University School of Medicine, St. Louis, MO USA; 5 Department of Psychology, University of Johannesburg, Johannesburg, South Africa; 6 Hope Center for Neurological Disorders, Washington University School of Medicine, St. Louis, MO USA; 7 Department of Pathology & Immunology, Washington University School of Medicine, St. Louis, MO USA; 8 Department of Physical Therapy, Washington University School of Medicine, St. Louis, MO USA; 9 Department of Occupational Science & Occupational Therapy, Washington University School of Medicine, St. Louis, MO USA; 10 Department of Occupational Science and Occupational Therapy, University of Toronto, Toronto, ON Canada

**Keywords:** Naturalistic driving, Preclinical Alzheimer disease, Global positioning system, Machine learning

## Abstract

**Background:**

Alzheimer disease (AD) is the most common cause of dementia. Preclinical AD is the period during which early AD brain changes are present but cognitive symptoms have not yet manifest. The presence of AD brain changes can be ascertained by molecular biomarkers obtained via imaging and lumbar puncture. However, the use of these methods is limited by cost, acceptability, and availability. The preclinical stage of AD may have a subtle functional signature, which can impact complex behaviours such as driving. The objective of the present study was to evaluate the ability of in-vehicle GPS data loggers to distinguish cognitively normal older drivers with preclinical AD from those without preclinical AD using machine learning methods.

**Methods:**

We followed naturalistic driving in cognitively normal older drivers for 1 year with a commercial in-vehicle GPS data logger. The cohort included n = 64 individuals with and n = 75 without preclinical AD, as determined by cerebrospinal fluid biomarkers. Four Random Forest (RF) models were trained to detect preclinical AD. RF Gini index was used to identify the strongest predictors of preclinical AD.

**Results:**

The F1 score of the RF models for identifying preclinical AD was 0.85 using *APOE* ε4 status and age only, 0.82 using GPS-based driving indicators only, 0.88 using age and driving indicators, and 0.91 using age, *APOE* ε4 status, and driving. The area under the receiver operating curve for the final model was 0.96.

**Conclusion:**

The findings suggest that GPS driving may serve as an effective and accurate digital biomarker for identifying preclinical AD among older adults.

## Introduction

Worldwide, around 50 million individuals are living with dementia, and this number is projected to increase to 152 million by 2050 [1]. Alzheimer disease (AD) is the most common form of dementia, accounting for 60 to 80% of cases [2–4]. Symptomatic AD impairs the cognitive and functional abilities required for performing activities of daily living, which can lead to hospitalizations, home care, and even institutionalization [5]. As a result, AD can place significant financial and emotional burdens on family members and society at large [6]. Given the growing socioeconomic impacts of AD, many studies have focused on the development of specific treatment strategies aimed at slowing down or even preventing the onset of symptomatic AD [7, 8]. However, these strategies may require AD to be diagnosed at an early stage before significant damage to the brain has occurred, when patients are still cognitively normal. Currently, the presence of AD brain pathology can be determined by molecular biomarkers obtained via imaging or lumbar puncture. However, the use of these methods is limited by cost, acceptability (i.e. willingness to participate in research or assessments involving cerebrospinal fluid (CSF) collection), and availability [9, 10]. Therefore, there is an increasing need for low-cost and low-burden methods to make an early diagnosis of AD.

Preclinical AD is the period during which early AD brain changes are present but cognitive symptoms have not yet manifest [11]. The preclinical phase of AD can include subtle cognitive changes, which may go unnoticed. However, emerging evidence suggests that these changes impact complex behaviours that involve both cognitive and functional abilities such as spatial navigation and driving. Since subtle cognitive changes may precede a clinical diagnosis of dementia by up to 20 years, tracking and recording navigational and driving abilities could potentially enable earlier identification of individuals with AD [12, 13]. A few studies have explored the utility of spatial navigation deficits as a marker for early and preclinical AD and discussed its high specificity for identifying at-risk individuals [13, 14]. This work, however, focuses on the potential of driving, the primary means of transportation for older adults, to detect underlying pathophysiology in preclinical AD.

Road tests and driving simulators are commonly used for assessing fitness-to-drive in clinical and healthy populations [15]. These standardized assessments, however, only measure performance in site-specific controlled conditions and do not assess daily driving behaviour in naturalistic settings. In addition, these assessment methods are methodologically limited by objectivity, availability, generalizability, and cost-effectiveness [15, 16]. To overcome these limitations, the field of driving research has shifted to collecting naturalistic outcomes using global positioning system (GPS) devices that can be installed in a participant’s personal vehicle.

Several studies have adopted a naturalistic approach and showed that everyday driving behaviour is associated with symptomatic AD. These studies show that drivers with symptomatic AD are more likely to drive shorter distances, visit fewer unique destinations, and have a smaller driving space compared to cognitively intact drivers [17, 18]. However, only a few studies, to date, have explored the impact of preclinical AD on driving behaviour. To our knowledge, our group is the first to use GPS to assess naturalistic driving behaviour among older drivers with preclinical AD [19]. In earlier work, we reported findings on a cross-sectional study that examined driving behaviour in a small sample (*n* = 20) of cognitively intact drivers with and without preclinical AD. Later, we reported results of the extension of the data collection and evaluated driving behaviour changes over a 2.5-year period [20]. Most recently, we presented findings of a proof-of-concept study showing that preclinical AD can be identified by evaluating driving behaviours [21]. In discussing the results of that study, we noted that future research aimed at using everyday driving as a behavioural marker of AD should explore additional statistical modelling techniques to determine optimal combinations of variables [21].

The objective of this paper is to use machine learning techniques to test the ability of GPS data to distinguish persons with and without preclinical AD, defined using cerebrospinal fluid, in a cohort of cognitively intact older adults from a longitudinal driving study. Specifically, we use machine learning to investigate the driving performance and driving space of older adults with and without preclinical AD. Further, we use feature selection to identify indicators that were the strongest discriminators of preclinical AD. This data-driven approach provides a foundation for the development of a novel neurobehavioural biomarker of AD.

## Methods

### Participants

Participants were enrolled in longitudinal studies on ageing and dementia conducted at the Washington University Knight Alzheimer Disease Research Center and in a longitudinal driving study (R01AG056466). Participants who met the following criteria were included in the study: (1) were age 65 years or older, (2) had normal cognition at a clinical assessment that included assignment of the Clinical Dementia Rating™ (CDR™) [22], (3) underwent CSF collection, (4) possessed a valid driving licence, and (5) drove at least weekly, on average. Participants provided written informed consent, and all study procedures were approved by the Washington University Human Research Protection Office.

### CSF biomarkers and *APOE* genotyping

CSF was collected as previously described [23]. CSF Aβ42 and Aβ40 analytes were measured using the Lumipulse G1200 automated assay system (Fujirebio, Malvern, PA). Aβ42/Aβ40 < 0.0673 is highly concordant with positive status by amyloid positron emission tomography (PET) [24] and was used to identify individuals with preclinical AD. Taqman genotyping of rs7412 and rs429358 was used to determine *APOE* genotype [25].

### GPS data collection

A GPS data logger (G2 Tracking Device™, Azuga Inc, San Jose, CA) was installed into the onboard diagnostics-II (OBD-II) port of each participant’s vehicle. This data logger, together with custom software, comprises the Driving Real-World In-Vehicle Evaluation System (DRIVES) [20, 21, 26]. The DRIVES recorded date, time, latitude and longitude coordinates, and speed every 30 s when a vehicle was driven. For each participant, 1 year of GPS driving data from January 1, 2019, to December 31, 2019, was included for analysis in this study. The 1-year study period was selected to account for seasonal variability in travel behaviours.

### GPS-based driving behaviour measures

A comprehensive set of GPS-based indicators that describe everyday driving behaviour were examined. The indicators describe either the *driving performance* or *driving space* for each participant. Driving performance indicators capture speed, acceleration, and vehicle jerk characteristics, as well as aggressive driving incidents (e.g. hard braking), while driving space indicators capture the spatiotemporal characteristics of outdoor excursions. To select these indicators, we searched the literature for articles that used GPS technology to evaluate driving performance and measure life-space [20, 27, 28]. We selected the indicators that were most frequently used in these articles. The definitions of our proposed GPS-based indicators are presented in Table 1.

**Table 1:** Description of the GPS-based driving indicators

Characteristics	Indicator	Abbreviation	Description
**Driving space**	Average trip distance	TripDist	The average distance travelled in each trip. TripDist is computed by taking the average of all the trips that a participant has made during the study period.
	Total travelled distance	TotalDist	The total distance travelled during the study period.
	Number of trips	nTrips	The total number of trips made during the study period. The trips are also placed into five subgroups: (1) trips with a distance smaller than 1 mi, (2) trips with a distance between 1 and 5 mi, (3) trips with a distance between 5 and 10 mi, (4) trips with a distance between 10 and 20 mi, and (4) trips with a distance of more than 20 mi.
	Radius of gyration	Rg	Typical distance travelled by an individual, computed using [29]: ${r}_g=\sqrt{\frac{1}{N}\sum \limits_{i\epsilon L}{n}_i{\left({r}_i-{r}_{cm}\right)}^2}$ where L is the set of destinations by the individual, *r*_*i*_ is the latitude and longitude coordinates of location *i*, *n*_*i*_ is the visitation frequency of location *i*, $N=\sum \limits_{i\ \epsilon L}{n}_i$ is the total number of visits of the individual, and *r*_*cm*_ is the center of mass of the visited destinations.
	Entropy	S	The degree to which a participant’s trip destinations are random (i.e. unpredictable) in space and time [30]. S is assessed over the entire period of the study.
	Number of night trips	nNightTrip	The average number of trips made after sunset.
	Number of unique destinations	nUniqDest	The total number of distinct destinations that an individual visited during the entire study period.
**Driving performance**	Number of hard brakes per mile	nHardBrake	The average number of events with a deceleration rate of above 8 miles per hour in 1 s per mile.
	Number of sudden acceleration per mile	nSuddenAcc	The average number of events with an acceleration rate of above 8 miles per hour in 1 s per mile.
	Overspeed	OverV	The average number of trips with a speed of 6 miles per hour above the posted speed limit.
	Underspeed	UnderV	The average number of trips with a speed of 6 miles per hour below the posted speed limit.
	Average speed	avgV	The average speed of trips.
	Average acceleration	avgA	The average acceleration of trips.
	Average jerk	avgJ	The average jerk of trips. Jerk is the rate of change of acceleration [31]; that is, more abrupt brake actions or accelerations lead to higher jerk values.

### Machine learning and statistical analyses

All analyses were performed in Python. Data from participants with CSF biomarkers within 2 years of January 1, 2019, were selected. We regarded the prediction model for preclinical AD as a machine learning problem with a binary output, where class 0 included participants without preclinical AD (CSF Aβ42/Aβ40 ≥ 0.0673) and class 1 included participants with preclinical AD (CSF Aβ42/Aβ40 < 0.0673). Random Forest (RF) classifier, a robust tree-structured machine learning algorithm, was used for this problem. RFs were selected because they have proven to outperform classical machine learning models in terms of accuracy and are more interpretable than deep learning models [32]. In addition, they are effective at handling high-dimensional data and are robust to outliers [33]. We trained four RF-based models with four sets of input variables: (1) age and *APOE ε4* status (carrier or non-carrier), (2) driving features only, (3) driving features and age, and (4) driving features, age, and *APOE ε4* status. Predictive features of preclinical AD were ranked according to importance using RF Gini index, which is a method that ranks features based on how much they contribute to the model. All models were trained on 70% of the data and tested on the remaining 30% of the data. Note that each month record for each participant was considered an independent data point. To achieve the best performance, the models’ specifications were selected based on incremental hyperparameter tuning; that is, each model was trained on many hyperparameters on training data, and then models that were performing better were selected. For performance evaluation, the precision, recall, and F1 score were calculated and compared across the four models. In this problem, we define preclinical AD to be positive class and no preclinical AD to be negative class. Given these definitions, precision measures the number of true positives (i.e. preclinical AD) divided by the total number of predicted positives. That is, the ratio of predicted preclinical AD subjects who truly have preclinical AD over all subjects predicted to have preclinical AD. Recall, however, measures the ratio of true-positive cases over the total number of true-positive and false-negative cases. Finally, to compare the performances of the models, the F1 score is used, which combines precision and recall into a single number by taking their harmonic. In addition, in a similar approach to previous studies that tested the ability of a novel biomarker to distinguish between clinical groups [34, 35], a receiver operating curve (ROC) was generated and its area under the curve (AUC) was computed for each model. Models’ performance metrics are reported on the test set, and 1000 bootstrapped samples were used to calculate 95% confidence intervals.

## Results

### Sample characteristics

Participant characteristics are presented in Table 2. The two groups did not differ significantly with regard to sex, race, or years of education level. The descriptive statistics and effect sizes of driving indicators are tabulated in Table 3.

**Table 2:** Sample characteristics

	Without preclinical AD (*n* = 75)	With preclinical AD (*n* = 64)
**Age, years**	75.7 ± 4.8	79.1 ± 4.90
**APOE ε4+ carrier, %**	30	33
**Education, years**	16.4 ± 2.3	16.5 ± 2.43
**Sex, % female**	51%	47%
**Race**^**a**^**, % White**	84%	92%

^a^The sample includes only Blacks and Whites

**Table 3:** Driving indicators’ descriptive statistics and effect sizes using Cohen’s d

	Without preclinical AD (*n* = 75)	With preclinical AD (*n* = 64)	Cohen’s d^**a**^
**TripDist, km**	8.1 ± 2.6	7.9 ± 2.8	0.07
**TotalDist, km**	891.5 ± 371.4	787.5 ± 368.8	0.28
**nTrips**	113.7 ± 40.4	103.3 ± 41.9	0.25
**Rg, km**	67.0 ± 98.8	44.0 ± 63.5	0.27
**S**	3.97 ± 0.5	3.84 ± 0.5	0.26
**nNightTrip**	53.2 ± 19.4	46.43 ± 19.7	−0.35
**nUniqDest**	38.2 ± 13.0	34.8 ± 13.9	0.25
**nHardBrake**	0.027 ± 0.04	0.022 ± 0.02	0.15
**nSuddenAcc**	0.039 ± 0.05	0.034 ± 0.03	0.12
**OverV**	0.07 ± 0.08	0.06 ± 0.05	−0.15
**UnderV**	0.20 ± 0.12	0.23 ± 0.15	−0.22
**avgV, m/s**	8.03 ± 1.87	8.04 ± 1.83	−0.01
**avgA, m/s**^**2**^	2.84 ± 0.35	2.79 ± 0.41	0.15
**avgJ, m/s**^**3**^	1.46 ± 0.16	1.39 ± 0.20	0.39

^a^Effect sizes (Cohen’s d) of 0.2 are considered small, 0.5–0.6 are considered medium, and 0.8 are considered large

*Abbreviations*: *TripDist* average trip distance, *TotalDist* total travelled distance, *nTrips* number of trips, *RG* radius of gyration, *S* entropy, *nNightTrip* number of night trips, *nUniqDest* number of unique destinations, *nHardBrake* number of hard brakes per mile, *nSuddenAcc* number of sudden accelerations per mile, *OverV* overspeed, *UnderV* underspeed, *avgV* average speed, *avgA* average acceleration, *avgJ* average jerk

### RF models

The precision, recall, and F1 score for each model are presented in Table 4. The performance of the model for predicting preclinical AD improved with the addition of age alone, and age and *APOE4 ε4* status. The final model with all the features achieved an F1 score of 0.91 (95% CI 0.893–0.937). The model was correct in identifying 96% of individuals with preclinical AD (by the precision measure). Among all participants with preclinical AD, the model correctly identified 88% (by the recall measure).

**Table 4:** Assessment of the model performance on the test set. Values in parentheses represent 95% confidence intervals

Model inputs	Precision	Recall	F1 score	AUC
**Age and** ***APOE ε4*** **status**	0.84 (0.802–0.875)	0.79 (0.770–0.861)	0.85 (0.833–0.852)	0.88 (0.861–0.927)
**Driving features**	0.89 (0.862–0.917)	0.76 (0.716–0.796)	0.82 (0.794–0.840)	0.82 (0.782–0.932)
**Driving features and age**	0.94 (0.909–0.959)	0.84 (0.794–0.876)	0.88 (0.858–0.906)	0.94 (0.881–0.962)
**Driving features, age, and** ***APOE ε4*** **status**	0.96 (0.939–0.981)	0.88 (0.837–0.912)	0.91 (0.893–0.937)	0.96 (0.903–0.981)

**Fig. 1:**
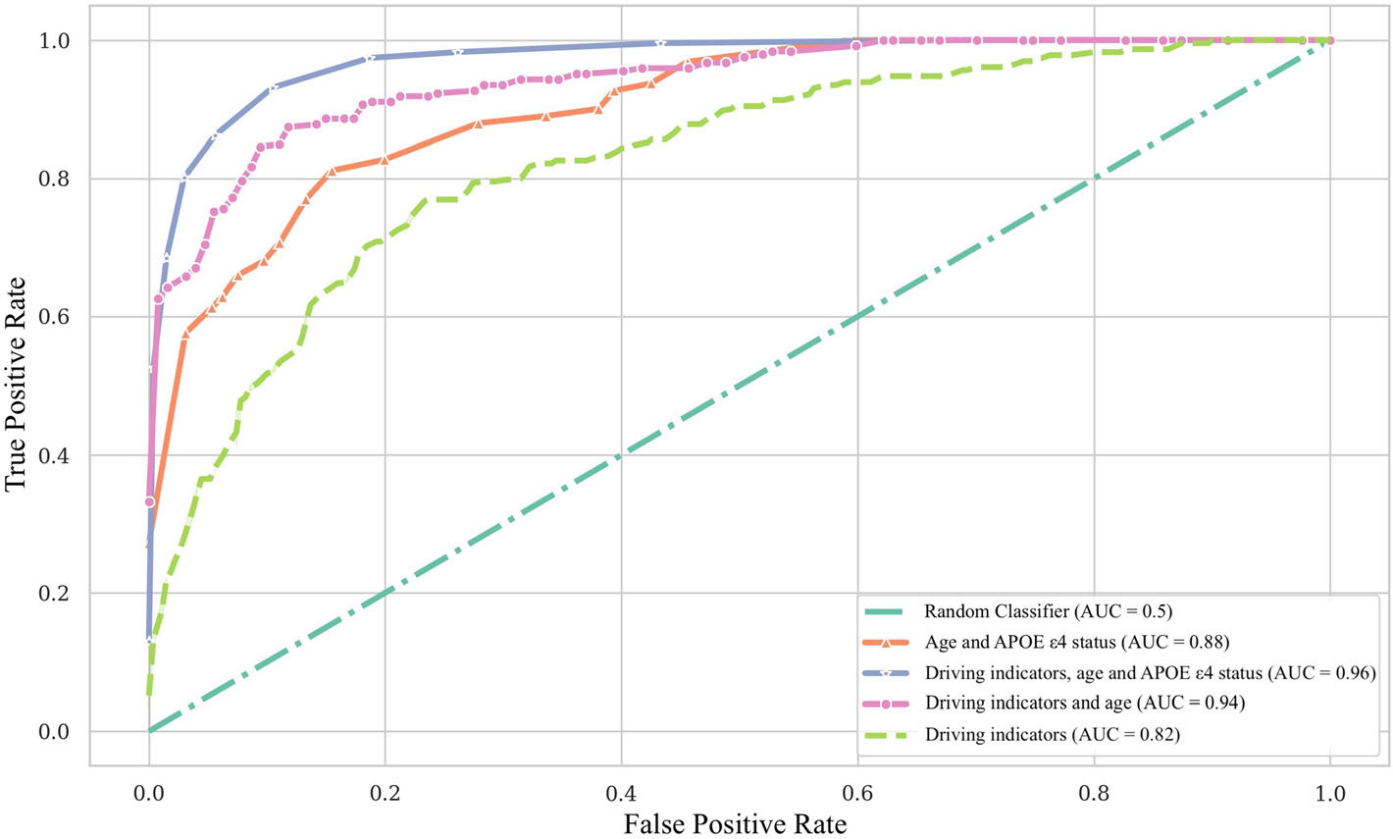
The final area under the receiver operating curves (AUC) for each model. Legends show the AUC as each feature is added to the model

The ROC area under the curve (AUC) for predicting preclinical AD from driving features was 0.82 (95% CI 0.782–0.932) (Fig. 1) and improved with the addition of age alone to 0.94 (95% CI 0. 881–0.962), and age and *APOE* ε4 status 0.96 (95% CI 0.0.903–0.981) (Fig. 1).

### Driving indicator importance

**Fig. 2:**
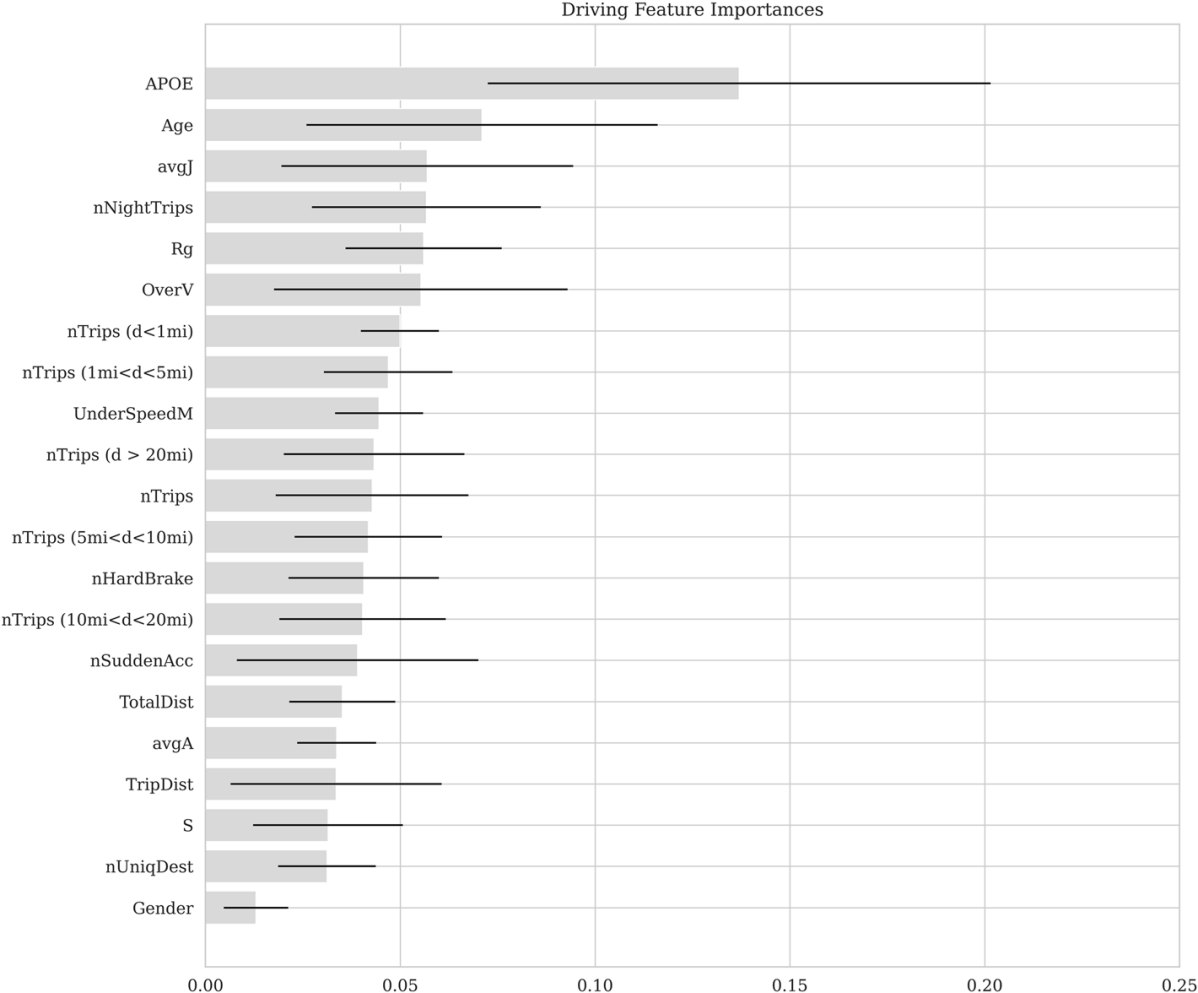
Importance ranking of all features

The ranked importance scores of the features are presented in Fig. 2. The five most important features were *APOE4 ε4* status, age, average jerk, number of night trips, and radius of gyration.

## Discussion/conclusion

Our findings suggest that driving may serve as an effective and accurate digital biomarker for identifying preclinical AD among older adults. In fact, the increasing availability of GPS devices is creating an environment where the naturalistic driving behaviours of older drivers are continuously monitored. This trend may make the analysis of naturalistic driving a non-invasive, unobtrusive, and low-cost solution for identifying individuals who are likely to have preclinical AD.

A novel finding of our study is the application of machine learning methods to a large dataset of GPS driving trajectories to predict preclinical AD. To provide an inclusive view of the driving behaviours of older adults with and without preclinical AD, we created a comprehensive set of GPS-based indicators describing daily driving performance and space. Using driving indicators alone, our predictive model achieved an average F1 score of 0.82 (95% CI 0.79–0.84), indicating the model’s high robustness and precision. The 0.89 precision score indicates that the model correctly predicts preclinical AD 89% of the time. The 0.76 recall score indicates that among the participants with preclinical AD, the model correctly identified 76%. Furthermore, compared to this model, the model with age and *APOE ε4* status alone achieved a higher F1 score and recall score, but a lower precision score. The higher precision score of the model with driving indicators is indicative of the model’s lower false-positive rate (i.e. predicted preclinical AD, but the subject did not have preclinical AD). This may be, at least partially, due to the fact that driving features reflect actual changes that are occurring on an individual basis due to the biological presence of AD, while APOE4 are risk factors for developing AD. In addition, it is important to note that the age and *APOE ε4* status model requires only two predictors as opposed to more than ten predictors in the driving behaviour model. It is, however, more invasive and less accessible because it requires APOE genotype testing.

The predictive power of the model with driving indicators was improved by including age and *APOE* ε4 status [21]. In fact, the predictive model with driving behaviour and age alone achieved an F1 score of 0.88 (95% CI 0.86–0.91), and the model with driving behaviour, age, and *APOE ε4* status achieved an F1 score of 0.91 (95% CI 0.89–0.94). This improvement is unsurprising since age and *APOE* ε4 are among the strongest risk factors for AD [36]. Others have shown that the ability of novel AD biomarkers to predict preclinical AD (generally indicated by abnormality of PET or CSF amyloid biomarkers) may be improved by including age and *APOE ε4* status in models [34, 37, 38].

Overall, although the model with driving behaviour, age, and *APOE ε4* status achieved the highest performance, the model with driving indicators and age alone is the highest performing non-invasive and accessible choice. This finding is important because, given the small size and ease of installation of vehicle GPS trackers, they can be mailed to clinics and individuals, allowing widespread use in different environments (i.e. urban and rural). It is also important to note that APOE genotype testing, although invasive, is becoming more accessible through new commercial platforms such as 23 and me [39]. Therefore, GPS driving in combination with age alone or age and *APOE* genotype can be used as an accurate, easily implementable, and cost-effective biomarker to identify preclinical AD.

Another key finding of our study is that the importance ranking of all the features. The results confirmed that *APOE* ε4 status and age are the two most important features for predicting preclinical AD. Interestingly, the most important driving feature was jerk, which is a measure of the smoothness or abruptness of driving. Although studies on driving behaviour in naturalistic settings have used vehicle jerk to identify unsafe and aggressive driving behaviour [31, 40], no study to date has examined vehicle jerk to identify AD. Furthermore, the top five most important features consisted of two features describing driving performance (average jerk and over speeding) and three features describing driving space (total number of night trips, radius of gyration, and number of trips shorter than 1 mi). Thus, our results suggest that both driving space and driving performance features have to be considered simultaneously to identify preclinical AD.

Because the decline in CSF Aβ42 is one of the earliest pathological events in AD [41], preceding the appearance of dementia symptoms by up to 20 years, we used Aβ42/Aβ40 as our marker of neuropathological AD among our cognitively normal participants. However, other changes in total tau and phosphorylated tau, and neurodegeneration, occur subsequently in the disease course, suggesting that future research should examine how driving behaviours predict the presence of abnormalities in other CSF biomarkers such as tau, phosphorylated tau181, phosphorylated tau217, and neurofilament light. Furthermore, recent advances in the development of plasma AD biomarkers have led to newly available blood tests for abnormality of AD-related proteins [42], and these blood tests may ultimately become widely used in clinical practice to diagnose AD. Machine learning methods like those used here should also be applied to determining the optimal combination of driving behaviours to identify and predict blood-based AD diagnoses.

## Limitations

The findings of this study should be considered in light of a number of inherent limitations, which can be addressed in future work. First, although most of our participants were the sole drivers of their vehicles, no automatic method was available to identify drivers and, in fact, friends, spouses, and family members may have made a small number of trips. Second, all the participants were from the greater St. Louis metropolitan area, and thus, the findings may not be generalizable to other regions. Third, future studies with a larger sample should further investigate the role of sociodemographic attributes including sex, race, income, and education level in the driving patterns, since these attributes may contribute to various factors, including social and cultural norms affecting daily driving behaviour [43]. In addition, our sample’s racial make-up, similar to that of the surrounding areas, only consists of Blacks and Whites, limiting the generalizability of the findings. Thus, future research with participants of other races and ethnicities is warranted. Finally, since repeated measures were treated as independent measures, future studies should further incorporate the longitudinal nature of the data by exploring machine learning methods that handle the correlated features from participants over time such as repeated measures random forest.

## Data Availability

The datasets used and/or analysed during the current study are available from the corresponding author on reasonable request.

## Abbreviations

AD: Alzheimer disease
ML: Machine learning
CSF: Cerebrospinal fluid
RF: Random Forest
ROC: Receiver operating curve
AUC: Area under the curve
